# Prenatal Detection of Aneuploidy and Imbalanced Chromosomal Arrangements by Massively Parallel Sequencing

**DOI:** 10.1371/journal.pone.0027835

**Published:** 2012-02-28

**Authors:** Shan Dan, Fang Chen, Kwong Wai Choy, Fuman Jiang, Jingrong Lin, Zhaoling Xuan, Wei Wang, Shengpei Chen, Xuchao Li, Hui Jiang, Tak Yeung Leung, Tze Kin Lau, Yue Su, Weiyuan Zhang, Xiuqing Zhang

**Affiliations:** 1 Department of Perinatal Medicine, Beijing Obstetrics and Gynecology Hospital – Capital Medical University, Beijing, China; 2 Beijing Genomics Institute at Shenzhen, Shenzhen, China; 3 Fetal Medicine Unit, Department of Obstetrics and Gynecology, Prince of Wales Hospital, The Chinese University of Hong Kong, Hong Kong SAR, China; Institut Jacques Monod, France

## Abstract

Fetal chromosomal abnormalities are the most common reasons for invasive prenatal testing. Currently, G-band karyotyping and several molecular genetic methods have been established for diagnosis of chromosomal abnormalities. Although these testing methods are highly reliable, the major limitation remains restricted resolutions or can only achieve limited coverage on the human genome at one time. The massively parallel sequencing (MPS) technologies which can reach single base pair resolution allows detection of genome-wide intragenic deletions and duplication challenging karyotyping and microarrays as the tool for prenatal diagnosis. Here we reported a novel and robust MPS-based method to detect aneuploidy and imbalanced chromosomal arrangements in amniotic fluid (AF) samples. We sequenced 62 AF samples on Illumina GAIIx platform and with averagely 0.01× whole genome sequencing data we detected 13 samples with numerical chromosomal abnormalities by z-test. With up to 2× whole genome sequencing data we were able to detect microdeletion/microduplication (ranged from 1.4 Mb to 37.3 Mb of 5 samples from chorionic villus sampling (CVS) using SeqSeq algorithm. Our work demonstrated MPS is a robust and accurate approach to detect aneuploidy and imbalanced chromosomal arrangements in prenatal samples.

## Introduction

Chromosomal abnormalities occur in 1 of 160 live births [1].The risk of giving birth to a child with chromosomal abnormalities, especially Down syndrome (OMIM# 190685), increases throughout a woman's reproductive years [2], [3]. Prenatal diagnosis of fetal chromosomal abnormalities is the most common indication for invasive prenatal testing. The prevalence of chromosomal abnormalities in fetuses with aneuploidy accounts for 6–11% of all stillbirths and neonatal deaths [4], [5]. Consequently, screening and diagnostic programs to detect the most common trisomies in live born infants are well established [6]. Currently, G-band karyotyping and several molecular genetic methods including multiplex ligation-dependent probe amplification (MLPA), fluorescence in situ hybridization (FISH), quantitative fluorescent PCR (QF-PCR) and microarray-based comparative genomic hybridization (arrayCGH) have been well established for prenatal diagnosis of chromosomal abnormalities in clinical labs [7], [8]. Although these testing methods have been proved to be highly reliable, the major limitation remains restricted resolution or can only achieve limited coverage on the human genome at one time [9], [10].

To overcome these limitations, in this study we have developed a new method based on massively parallel sequencing (MPS) platform to detect fetal chromosomal abnormalities, which is independent of particular genetic markers and cell culture in medical practice. We directly sequenced 62 DNA samples extracted from uncultured amniotic fluid (AF) of pregnant women. After statistical analysis, we can clearly detect numerical chromosomal abnormalities among 46 chromosomes. The whole process only takes 7 days and the results were validated by full karyotyping analysis. Subsequently, we investigated the presence of copy number variations among 5 prenatal samples previously identified by arrayCGH. We demonstrated that with different whole genome sequencing coverage MPS platform could be applied for identification of aneuploidy and imbalanced chromosomal arrangements, and those approaches are more sensitive and effective compared with conventional methods.

## Results

### Detection of Fetal Aneuploidy

**Figure 1:**
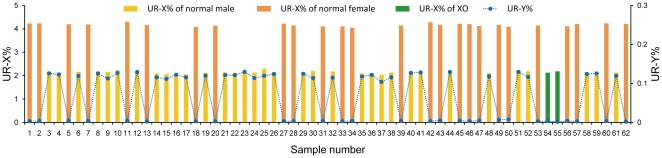
UR-X% and UR-Y% of 62 amniotic fluid samples.

**Figure 2:**
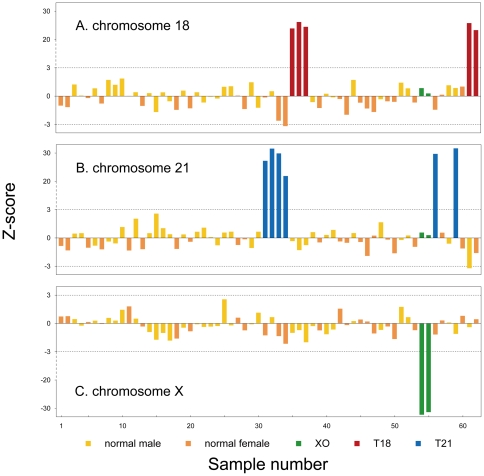
z-scores of 62 AF samples for chromosome 21,18 and X determined by MPS. Broken lines indicate the z score cut-off value of ±3 for trisomy 21 and trisomy 18. All 11 trisomy cases have a z-score value greater than 3 and the 2 cases of Tuner syndrome have a z-score value less than minus 3.

In this study, the 32 test cases were used for sequencing analysis under double-blind conditions. For all 62 AF samples (30 normal controls and 32 test cases), we obtained 0.55∼3.28 million 35-bp single-end reads (or 19.25 Mb∼114.80 Mb sequencing data) which corresponds to 0.006∼0.038× human genome depth. Consequently, 0.20∼1.15 million unique reads (UR) were obtained for each sample (Table 1). For each chromosome, the number of unique reads was counted and the UR% was calculated. The complete set of UR% for 62 samples is listed in Table S1. UR% for Chromosome X, Y also showed in Fig. 1. The gender of each sample can be determined from UR% of Chromosome X, Y. For 30 normal control samples, the mean and standard deviation (SD) of UR% for each chromosome were calculated. Then the *z-scores* of each of the chromosomes, except the Y chromosome, for each of the normal controls and test cases were calculated according to the formula described in *Materials and methods* (Table S2). From 32 test cases, 5 cases had a z-score>20 for chromosome 18 (indicating Trisomy 18), 6 cases had a z-score>20 for chromosome 21 (indicating Trisomy 21), and 2 cases had a z-score←20 for chromosome X (indicating XO). All the other chromosomes had z-scores within ±3 for all 32 test cases (indicating euploid chromosomes) (Fig. 2, Table. S1). The detection of fetal aneuploidy based on sequencing analysis agreed with karyotyping results.

**Table 1:** Raw data and UR% of chromosome 21, 18, 13, X, Y of 62 AF sample.

Sample No.	Labrary No.	Fetal Karyotype	DNA Amount (ng)	Raw Data (reads)	Unique Data (reads)	UR-21%	UR-18%	UR-13%	UR-X%	UR-Y%
1	92H13	46XX	100	1 387 306	531 327	1.26909	2.91948	3.68963	4.22414	0.00320
2	08H9	46XX	100	1 649 958	622 928	1.26638	2.90997	3.64248	4.22633	0.00385
3	09H10	46XY	100	1 474 317	552 990	1.29496	3.05087	3.91725	2.16279	0.12586
4	10H11	46XY	100	1 501 313	577 494	1.29664	2.98046	3.74255	2.12764	0.12295
5	11H12	46XX	100	1 341 688	514 366	1.26525	2.96754	3.68026	4.18671	0.00447
6	12H14	46XY	100	1 315 342	502 804	1.26968	3.02663	3.77026	2.15392	0.11913
7	13H15	46XX	100	1 520 307	589 707	1.26164	2.93281	3.70743	4.17665	0.00407
8	15H18	46XY	100	1 548 855	589 173	1.27874	3.07974	3.94434	2.16982	0.12560
9	17HCGY	46XY	100	749 354	309 033	1.27818	3.04466	3.87758	2.12275	0.12264
10	19H2	46XY	100	1 929 900	744 124	1.30932	3.08860	3.90929	2.21683	0.12699
11	21H4	46XX	100	3 285 106	1 244 513	1.26613	2.97819	3.82873	4.29276	0.00410
12	25H9	46XY	100	1 575,322	667 903	1.32747	3.00182	3.86819	2.16540	0.12929
13	27H1	46XX	100	947 196	377 647	1.27924	2.91754	3.67009	4.09139	0.02304
14	31H6	46XY	100	1 248 950	476 205	1.29776	2.98863	3.57304	2.05458	0.13776
15	32H7	46XY	100	693 356	268 692	1.34987	2.88137	3.58328	2.01383	0.12840
16	33H8	46XY	100	929 156	371 080	1.31239	2.99262	3.70971	2.05805	0.13825
17	34H9	46XY	100	1 647 104	646 180	1.30010	2.94190	3.62840	2.01244	0.13263
18	40H6	46XX	100	728 707	278 944	1.26190	2.88158	3.41323	4.01586	0.01972
19	42H8	46XY	100	2 021 819	829 302	1.29856	3.00795	3.82840	2.08272	0.13578
20	43H9	46XX	100	685 181	270 538	1.27967	2.90236	3.52557	4.06486	0.02735
21	44H10	46XY	100	993 450	400 726	1.30688	3.00180	3.72499	2.09894	0.13550
22	45H11	46XY	100	636 527	250 931	1.30315	2.93308	3.64762	2.08464	0.13709
23	46H12	46XY	100	1 010 133	399 849	1.30249	2.96512	3.72216	2.09129	0.14480
24	47H13	46XY	100	764 726	301 493	1.28494	2.95828	3.58284	2.08827	0.13466
25	48H1	46XY	100	3 003 505	1 149 789	1.29728	3.03621	3.86358	2.27746	0.11976
26	54H7	46XY	100	1 561 427	589 196	1.29923	3.03974	3.85305	2.12900	0.12424
27	56H9	46XX	100	2 322 635	885 707	1.27119	2.98022	3.78613	4.21618	0.00339
28	57H10	46XX	100	1 278 926	512 310	1.29219	2.89961	3.57791	4.06414	0.02264
29	60H14	46XY	100	1 618 924	616 422	1.26456	3.06495	3.95038	2.13555	0.12475
30	62H16	46XY	100	661 358	263 458	1.30875	2.89420	3.73532	2.17530	0.12791
31	61HWMH	47XX+21	100	833 041	340 693	1.85651	2.98157	3.71009	4.05644	0.01967
32	72HT21	47XY+21	100	766 103	311 852	1.93585	3.01072	3.82617	2.13627	0.12955
33	74H3	47XX+21	100	1 127 241	458 743	1.91087	2.81705	3.54033	4.03080	0.02180
34	81HWMH	47XX+21	100	650 242	273 303	1.73434	2.77714	3.39257	3.96812	0.02378
35	02H2	47XY+18	90	1 091 649	429 544	1.28206	4.39862	3.64852	2.05544	0.12828
36	06H7	47XY+18	100	1 851 667	706 651	1.26689	4.54298	3.71881	2.10174	0.12198
37	82HCXP	47XY+18	100	503 179	204 295	1.27707	4.42742	3.78815	1.99858	0.11601
38	04H4	46XY	100	1 754 104	666 002	1.29819	2.94188	3.67957	2.12507	0.11662
39	05H6	46XX	100	2 434 717	898 966	1.27680	2.90456	3.57889	4.13297	0.00389
40	63H17	46XY	90.8	685 313	275 177	1.30970	2.98680	3.86915	2.04850	0.14064
41	65H4	46XY	100	1 202 799	473 140	1.32244	2.97185	3.70208	2.07402	0.14879
42	66H9	46XX	100	1 623 201	617 337	1.27888	2.96166	3.83680	4.27724	0.00340
43	67H10	46XX	100	811 894	327 382	1.28443	2.85660	3.64566	4.10071	0.02474
44	68H11	46XY	100	6 381 643	2 455 268	1.29619	3.07979	3.96616	2.15150	0.12931
45	69H12	46XX	100	955 289	384 784	1.28384	2.93281	3.62723	4.14050	0.02339
46	70H13	46XX	100	833 449	328 863	1.25554	2.91215	3.66147	4.13212	0.02037
47	71H14	46XX	100	1 321 322	523 536	1.29943	2.87430	3.56537	4.04327	0.02636
48	73H2	46XY	100	705 325	277 705	1.32443	2.94017	3.72230	2.07342	0.12639
49	77H8	46XX	100	664 485	271 294	1.28422	2.95805	3.74686	4.10035	0.02101
50	83HSHD	46XX	100	551 560	223 988	1.26480	2.93632	3.66657	4.01227	0.02411
51	07H8	46XY	100	1 383 844	525 106	1.28203	3.06243	3.92035	2.23364	0.12950
52	94HLCH	46XY	100	888 892	509 386	1.29097	3.02639	3.87113	2.17399	0.11661
53	95HSCJ	46XX	100	863 603	498 346	1.26719	2.93932	3.74920	4.13408	0.00301
54	96HLLR	45XO	100	880 178	508 298	1.29727	3.02775	3.67776	2.11766	0.00315
55	97HHH	45XO	100	984 935	565 960	1.29161	2.99420	3.87572	2.17719	0.00166
56	98HMCM	47XX+21	100	525 892	303 307	1.89610	2.89344	3.47536	4.10475	0.00396
57	99HZQL	46XX	100	898 477	518 330	1.29705	2.98664	3.77655	4.19713	0.00251
58	118AXCR	46XX	99	1 132 088	440 245	1.27406	3.04603	3.82810	2.14335	0.12402
59	121ASSH	47XY+21	100	1 045 028	394 985	1.93628	3.02847	3.72698	2.07983	0.12557
60	125A22	46XX	100	1 217 046	469 095	1.26350	3.03755	3.79454	4.22857	0.00384
61	126AJYJ	47XY+18	100	1 207 389	471 223	1.22044	4.51952	3.77189	2.11895	0.11948
62	129AGJ	47XX+18	100	1 181 077	449 114	1.25358	4.37083	3.69461	4.20539	0.00178

**Figure 3:**
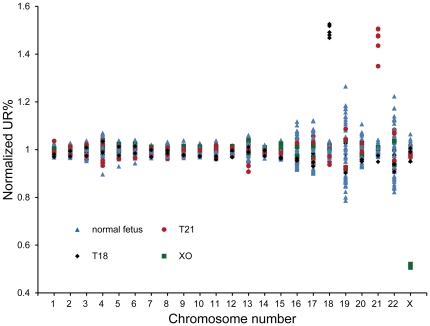
The normalized UR% value per chromosome between different samples. 11 trisomy samples have a normalized UR% value of 1.5 and 2 cases of 45 XO have a normalized UR% value of 0.5.

Normalized UR% values for all 22 autosomes and chromosome X were calculated (Fig. 3). 5 cases of trisomy 18, 6 cases of trisomy 21 and 2 cases of XO were successfully detected in the cohort of 32 test cases, which presented 100% of sensitivity and 100% of specificity.

### Detection of imbalanced chromosomal arrangements

To further validated our developed and optimized analysis method among cases with imbalanced chromosomal arrangements, we obtained 44∼70 M sequence tags for the 5 “known” samples with 1.4 to 2.3× whole genome depth. To detect segmental copy number variations, a sequence-based Matlab CNV detection package, SegSeq, with YH reference genome as comparative genome were used. By applying the SegSeq analysis, we detected microdeletions and microduplications in all 5 samples (Table 2). All the 5 chromosomal copy number variations ranged from 1.4 Mb to 37.3 Mb were validated by arrayCGH and were mapped precisely to the correct location. Four pregnancies were terminated after genetic detection and only one pregnant gave a live birth (Table S3).

**Table 2:** Pathogenic chromosomal copy number changes and imbalance rearrangements identified by SeqSeq analysis which have been validated by arrayCGH.

Array No.	Karyotype	arrayCGH	Segseq
A10071659	46,XY	deletion at 13q32.3q33.3(97091318∼106466788)	deletion at chr13: 97075576∼106516365(9.4 M)
A10021390	46,XY,der(5)t(5;18)(p13;q12.3)	deletion at 5p15.3∼p13.2(183931∼36816731);duplication at 18p12.3∼q23(39086755∼76067279)	deletion at chr5: 1∼36861739(36.9 M);duplication at chr18: 38768509∼76117152(37.3 M)
A10021383	46,XY,2q+	duplication at 2q36∼q37.3(230369496∼242444380)	duplication at chr2: 230288772∼242427293(12.1 M)
A11091844	46,XX	deletion at 3q29(197216353∼198770242)	deletion at chr3: 197372027∼198831453(1.4 M)
A10071710	46,XY	deletion at 22q11.21(17289032∼19636115)	deletion at chr22: 17396815∼20248184(2.8 M)

Two samples (A10021383, A10071659) were selected to further ascertain the results on the HumanOmni2.5 M chip (Illumina). CNV partition Algorithm plug-in, was used to detect copy number variations in these two samples. A duplication at 2q36∼q37.3 (230369496∼242444380) and a deletion at 13q32.3q33.3 (97091318∼106462788) were correctly detected, totally in concordance with the arrayCGH and sequencing analysis results.

## Discussion

Massively parallel sequencing has been reported only to apply in noninvasive prenatal diagnosis of trisomy 21, 18 and 13 based on cell-free fetal DNA, due to it is limited amount of fragmented fetal DNA [11]. This also makes noninvasive MPS-based prenatal diagnosis difficult to detect all the chromosomal aneuploidies and the sexual chromosome abnormality accurately. In this study, we demonstrated for the first time that combined MPS with powerful bioinformatics analysis method can accurately diagnosis fetal aneuploidy and imbalanced chromosomal structural abnormalities. In fact, this study reports the first retrospective use of MPS (so called next generation sequencing) for prenatal diagnostics of chromosomal imbalance rearrangements to date and shows that it is practically feasible on a large-scale prenatal diagnosis of fetal chromosomal abnormalities.

By establishing a normal control sequencing tag data set, we have been able to demonstrate this new approach only requires a minimum among of DNA materials (100 ng) to achieve the identification of aneuploidies with a ultra low sequencing coverage (0.01×). Comparing to the golden standard (G-Band karyotyping) in clinical practice, MPS has no time limitation. Also as long as 100 ng genomic DNA can be extracted from tissues at any gestational weeks, MPS can be performed and report all fetal aneuploidies in 7 days. If necessary, more sequence reads can be performed to detect whether microdeletion or microduplication exists in the fetal genome which may result in severe developmental retardation. In our study, tissues obtained from fetus, such as amniotic fluid, CVS and placenta can be analyzed without cell culture since 100 ng genomic DNA is sufficient for library preparation and sequencing. So it can be used for the research into the molecular mechanism of miscarriage, stillbirth and fetal death when tissues are difficult to culture. Furthermore, our validation study on the 5 arrayCGH samples show that when more sophisticated sequencing protocols and bioinformatics algorithms are applied to the analysis, it is possible to detect smaller size chromosomal copy number variations as well as complex rearrangements across the whole genome of the fetus, such as balanced chromosomal arrangements,structure variations or even single-gene disorders. With the application of third-generation sequencing system in clinical laboratories, such as Miseq/Illumina and Ion Torrent PGM/Life Technologies, the whole process will take less time and acceptable price. Thus, it is likely that MPS will play an increasingly important role in the future development of prenatal screening and diagnosis.

A potential weakness of the study was that in the figure of describing the ratio of unique reads in each chromosome, chromosome19 and 22 have a differently huge coefficient of variation because of their extremely high GC content and made the detection of trisomy19 and trisomy22 difficult. Further study will be set up to deal with these problems, for example the computational correction of GC content among chromosomes. Other chromosomal abnormalities, such as balanced translocation or incomplete aneuploidy caused by mosaics or partial duplication or deletion of a chromosome should, in principle, also be detectable. Further studies are required to determine the effectiveness of massively parallel genomic sequencing in detecting these rare aberrations. Another weakness for the new methodology was the starting materials for library construction; with the conventional Illumina library construction approach100 ng genomic DNA of fetus was required. To further reduce the risk to pregnancies it would be important to reduce the amount for AF or CVS samples, other library construction methods such as using in vitro transposition may be an alternative solution.

In conclusion, we have demonstrated the usefulness of massively parallel sequencing to detect fetal aneuploidy and imbalanced chromosomal abnormalities of genomic DNA in prenatal samples. In principle, massively parallel sequencing can also reveal other features of the genomic material from amniotic fluid such as histone modifications as well as epigenetic DNA methylation. With the rapid reduction of sequencing cost, we expect that the strategy described in this article will become a powerful tool in the detection of all kinds of chromosomal abnormalities in clinical settings.

## Materials and Methods

### Subject Enrollment and Sample Recruitment

The study was approved by the Institutional Review Board of Beijing Obstetrics and Gynecology hospital of the Capital Medical University. Informed consent was obtained from each participant.

A total of 32 pregnant women at a high risk of Down's syndrome were recruited as test cases from the Beijing Obstetrics and Gynecology hospital during the period of January to May 2010. Amniocentesis was applied at 19∼22th gestational week and standard G-band karyotyping analysis was performed. Another 30 euploid AF samples (20 with male fetus and 10 with female fetus) were included as normal control at the same hospital. To validate the sensitivity of analysis method for detection of microdeletion/microduplication, 5 chorionic villus sampling (CVS) samples validated by karyotyping and arrayCGH were recruited from the Prenatal Genetic Diagnosis Centre (PGDC) at Department of Obstetrics & Gynecology, Chinese University of Hong Kong.

### Sample Preparation and Sequencing

Genomic DNA was extracted from uncultured AF samples with Micro DNA Kit (Tiangen) and quantified with the Quant-iT dsDNA HS Assay Kit (Invitrogen). 100 ng genomic DNA from each sample was sheared into small fragments ranged from 100 to 400 bp with Bioruptor (Diagenode). After end-repair, “A”- overhanging and adapter-ligation, DNA fragments of 300 bp (±25 bp) in length were selected by 2% agarose gel electrophoresis and underwent 12 cycles of PCR with multiplex primers. PCR products were purified by Agencourt AMPure Kit (Beckman). Size distribution of the library was detected by Agilent Bioanalyzer DNA 1000 kit (Agilent Technologies) and the concentration was measured by quantitative PCR (qPCR). Libraries with different index tags were mixed in equal moles into a pool and sequenced with single-end 36 cycle multiplex sequencing on Illumina GAIIx platform.

For the 5 DNA samples from CVS tissues, sequencing libraries with the insert size of 500 bp (±25 bp) were prepared. Paired-end 100 cycle multiplex sequencing was performed on Illumina HiSeq 2000.

### Bioinformatics analysis

#### *z-score* for detection of fetal aneuploidy

35-bp single-end reads from 62 AF samples (30 normal controls and 32 test cases) were aligned against repeat-masked human genome build 36 (hg18) by ELAND. Unique reads (UR), which can be mapped to reference genome sequence without any mismatches or alternative positions, were used for further analysis. The UR percentage (UR%) of chromosome N (UR-N%, N denotes chromosome number) can be used as an indicator of fetal trisomy for autosomes. To satisfy central-limit theorem, 30 euploid samples were used as normal controls. The *z-score* of chromosome N (N stands for chromosome numbers) is calculated as,
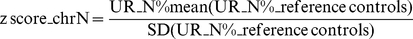
For autosomes, considering about the type I error rate (α) of 0.01, 3 was set as a cut-off value to determine the fetal trisomy.

#### SegSeq algorithm for detection of microdeletion/microduplication

We mapped the Illumina reads to the reference sequence of human genome (HG18, NCBI 36.3) by Short Oligonucleotide Analysis Package aligner (SOAP2) (http://soap.genomics.org.cn/) [12] with parameter about total allowed mismatches (-v 5), seed length (-s 40), minimal aligning length (-l 40) and insert DNA size enabled. Only unique reads were remained in following CNV analysis.

For the CNV detection, we employed a MATLAB packet, SegSeq (http://www.broadinstitute.org/cgi-bin/cancer/publications/pub_paper.cgi?mode=view&paper_id=182) [13], with YH Illumina reads [14] as reference control. Segments with copy ratio, calculated by SegSeq, less than 0.75 or greater than 1.25 were reported as variations. CNVs on critical regions of identified diseases would be an important signal for clinical screening/diagnose.

#### CNV Detection by arrayCGH and SNP typing array

Genomic chromosomal copy number variants (CNVs) were detected using a targeted high resolution 44 K oligonucleotide array specifically constructed for prenatal screening with the intention of targeting common trisomic aneuploidies and most known microdeletion and microduplication syndromes. This Fetal DNA chip included telomeric and pericentromeric regions, examining the genome to a resolution of 100 kb (http://www.fetalmedicine.hk/en/Fetal_DNA_Chip.asp) [15]. The Fetal DNA chip is specially (http://www.fetalmedicine.hk/en/Fetal_DNA_chip/Appendix_I.pdf) with most of the known common non-pathogenic CNVs regions removed. This chip provides a means to detect chromosomal aberrations with resolution of <100 Kb across the genome. The quality of the array was analysed using Agilent DNA analytics software and cases where the Derivative Log Ratio spread of the array was >0.25 were excluded from further data analysis. Data reporting variations in copy number were released after excluding known non-pathogenic chromosome copy number variants that have been listed at the Database of Genomic Variants.

To ascertain the accuracy of MSP-based CNV detection method, two CVS samples were analyzed with HumanOmni2.5-Quad Bead Chip according to Illumina manufacturer's protocol and CNV partition plug-in software was employed for DNA copy number analysis.

## Supporting Information

Table S1 **The UR% of each chromosome for 62 AF samples.**
(XLS)

Table S2 **The z-scores of each chromosome (except chr Y) for 62 AF samples.**
(XLS)

Table S3 **Outcome and clinical data of CUHK cases.**
(XLS)
